# Subradiant plasmonic cavities make bright polariton states dark

**DOI:** 10.1515/nanoph-2024-0058

**Published:** 2024-03-22

**Authors:** Ju Eun Yim, Zachary T. Brawley, Matthew T. Sheldon

**Affiliations:** Department of Chemistry, Texas A&M University, College Station, USA; Department of Materials Science and Engineering, Texas A&M University, College Station, USA; Department of Chemistry, 12377 University of California, Irvine, Irvine, CA, USA

**Keywords:** strong coupling, vibrational strong coupling, dark plasmon, vibropolaritons, bound states in the continuum, polariton chemistry

## Abstract

Nanostructured plasmonic surfaces allow for precise tailoring of electromagnetic modes within sub-diffraction mode volumes, boosting light–matter interactions. This study explores vibrational strong coupling (VSC) between molecular ensembles and subradiant “dark” cavities that support infrared quadrupolar plasmonic resonances (QPLs). The QPL mode exhibits a dispersion characteristic of bound states in the continuum (BIC). That is, the mode is subradiant or evanescent at normal incidence and acquires increasing “bright” dipole character with larger in-plane wavevectors. We deposited polymethyl methacrylate (PMMA) thin films on QPL substrates to induce VSC with the carbonyl stretch in PMMA and measured the resulting infrared (IR) spectra. Our computational analysis predicts the presence of “dark” subradiant polariton states within the near-field of the QPL mode, and “bright” collective molecular states. This finding is consistent with classical and quantum mechanical descriptions of VSC that predict hybrid polariton states with cavity-like modal character and *N−1* collective molecular states with minimal cavity character. However, the behaviour is opposite of what is standardly observed in VSC experiments that use “bright” cavities, which results in “bright” polariton states that can be spectrally resolved as well as *N−1* collective molecular states that are spectrally absent. Our experiments confirm a reduction of molecular absorption and other spectral signatures of VSC with the QPL mode. In comparison, our experiments promoting VSC with dipolar plasmonic resonances (DPLs) reproduce the conventional behavior. Our results highlight the significance of cavity mode symmetry in modifying the properties of the resultant states from VSC, while offering prospects for direct experimental probing of the *N−1* molecule-like states that are usually spectrally “dark”.

## 1 Introduction

Vibrational strong coupling (VSC) between an electromagnetic cavity and a molecular vibrational mode occurs when the resonant exchange of vibrational quanta can take place at a faster rate than energy dissipation into the environment. Strong coupling results in two modified states called the upper and lower “polariton”, separated in energy by the “vacuum Rabi splitting”. These new eigenstates of the coupled system have both light and matter character and are at the heart of the emerging research area termed “polariton chemistry”. VSC can also occur in the collective coupling regime when an ensemble of *N* molecules simultaneously couples with an infrared (IR) cavity mode. A series of studies have now reported that VSC not only modifies the spectroscopy of the coupled system, but may also impact the ground state potential energy landscape of the molecules [1], [2], [3], [4], [5], [6], energy transport [7], [8], and decay dynamics [9], [10].

Most VSC studies use Fabry–Pérot (F.P.) cavities that support a set of standing wave electromagnetic modes between two parallel mirror plates [1], [2], [3], [4], [5], [6], [7], [9]. However, mode volumes, V , of F.P. cavities are fundamentally bounded by the diffraction limit ∼ ${\left(\frac{\lambda }{2n}\right)}^{3}$ , where *n* is the refractive index of the F.P. cavity medium [11]. This constrains the single molecule coupling strength, $g_{s}\propto \sqrt{\frac{1}{V}}$ , and therefore requires greater *N* to increase the collective coupling strength, $g_{N}=\sqrt{N}g_{s}$ . Collective VSC, however, inevitably results in *N−1* states with nearly unperturbed energy compared to uncoupled molecules, often described as “dark” states [12]. That is, within the framework of the Tavis–Cummings model and comparable classical analogies, the upper and lower polaritons are the only two states in the coupled system of *N* + *1* states that exhibit significantly modified energy. All other *N*−*1* states correspond microscopically to combinations of individual molecular dipoles that provide an overall field amplitude that cannot exchange energy with the uncoupled cavity, so the *N−1* states take little character of the electromagnetic mode to which they are coupled. For an F.P. geometry, this means the *N−1* states have no overall dipole moment and therefore no signature in linear far-field spectra that are probed with planewave illumination, hence the designation as “dark”. Nonetheless these coupled *N−1* states are fundamentally distinct from the states associated with uncoupled molecules [12]. Currently, the role of the *N−1* states in polariton chemistry remains unclear [13], with many researchers arguing that their large number compared to the two polariton states entails that they dominate the physiochemical behavior of coupled systems [14], [15], [16].

Plasmonic nanocavities, in which sub-wavelength electromagnetic field enhancement is provided by resonant plasma oscillations of free carriers in metal nanostructures, offer an intriguing platform for studying VSC. Effective mode volumes are not constrained by the diffraction limit, allowing for strong coupling within the near-field of metal surfaces, even at the single molecule level [17], [18], [19]. Further, we recently demonstrated plasmonic geometries that promote collective VSC without angle-dependent frequency dispersion, so that molecular polaritons are observed independent of the relative orientation of the sample and probe geometry [20], [21]. Moreover, it has been discussed theoretically that the high local density of optical states (LDOS) and modifiable modal properties achievable using plasmonic nanostructures can provide enhancement of a variety of strong coupling effects compared to F.P. cavities [15], [21], [22], [23]. However, typical plasmonic modes suffer from lower quality factors (*Q*-factors) due to stronger radiative decay (i.e. scattering) and nonradiative decay (i.e. metal absorption) [11], [24], [25], [26]. Although it is challenging to modify intrinsic losses due to metal absorption [27], radiative loss can be minimized by taking advantage of “subradiant” plasmons. Subradiant plasmons, also called “dark” or “nonradiant” plasmonic modes, support electromagnetic field resonances with even symmetry (e.g. quadrupole and multipole modes), which leads to little or no interaction with free space radiation. Subradiant plasmons have higher *Q*-factors than that of “radiant” or “bright” plasmons due to suppressed radiative decay [28], [29], so they are expected to more easily reach the strong coupling regime according to the criteria that $\Omega_{R}>\frac{1}{2}\left(\gamma_{pl}+\gamma_{m}\right)$ , where Ω_*R*_ is the vacuum Rabi splitting an *γ*_*pl*_ and *γ*_*m*_ are the plasmon and molecular decay rates, respectively [30]. Note that Ω_*R*_ = 2*g*_*N*_ when there is no detuning between the plasmon frequency and the vibrational frequency. Despite these potential benefits, there are few reports studying strong coupling with subradiant plasmonic cavities, largely because these systems cannot be probed directly with far-field radiation in standard spectroscopy measurements. Using near-field techniques, such as scattering-type scanning near-field optical microscopy (s-SNOM) and electron energy loss spectroscopy (EELS), Rabi splitting from strong coupling has been observed in subradiant cavities [31], [32]. It is also theorized that “dark polaritons”, i.e. subradiant upper and lower polaritons, may be generated when higher-order multipole modes of spherical plasmonic nanoparticles strongly couple to quantum emitters [33], [34].

**Figure 1:**
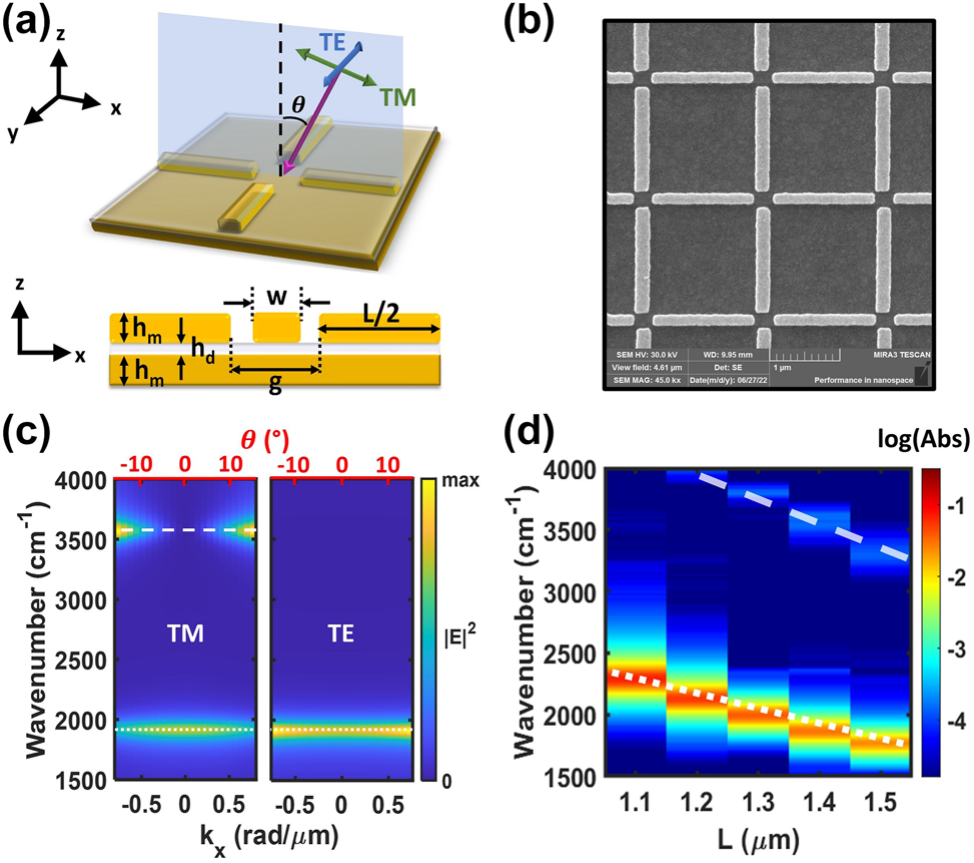
Far-field behaviour of Au nanorod arrays. (a) Schematic unit cell of the array composed of Au nanorods arranged with fourfold rotational symmetry. The electric field polarization of TE- and TM-polarized planewave as well as the corresponding wavevector are shown with blue, green, and pink arrows, respectively. The zenith angle (*θ*) of a non-normal planewave is also shown. The *xz*-cross section of the unit cell denoting the width (*w*), length (*L*), and height of Au rod (*h*_*m*_) is depicted below. The height of dielectric layer (*h*_*d*_) and gap width (*g*) are denoted. (b) Scanning electron microscope (SEM) image of an *L* = 1.4 µm array. (c) The calculated dispersion of the same array as a function of in-plane wavevector (*k*_*x*_) or angle of incidence (*θ*). The electric field intensity (|*E*|^2^) is normalized to the maximum value in the *x*- and *y*-axis range shown. (d) Experimental absorption with different lengths (*L*) of the rods. The absorption intensity is plotted on a log scale.

Here, we study collective vibrational strong coupling (VSC) between a molecular ensemble and subradiant plasmonic cavities. Our experiment is enabled by a cavity design (Figure 1) with the following features: first, the geometry supports a resonant mode with a strong dipole moment that provides “radiant” character, as well as a tailorable frequency resonance that can be tuned to the carbonyl stretch (C=O) of a deposited polymethyl methacrylate (PMMA) polymer thin film. We observe unambiguous collective VSC when probing this radiant, dipolar mode with far-field IR spectroscopy. Second, the geometry also supports a spectrally isolated subradiant plasmonic mode with a strong quadrupole moment and an even higher *Q*-factor. The resonant frequency of the subradiant mode can also be tuned to the carbonyl stretch (C=O) of deposited PMMA. Further, although neither cavity mode exhibits angle-dependent frequency dispersion, the subradiant quadrupolar mode gains increasing dipole character when probed at oblique angles. This angle-dependent transition from subradiant to radiant mode behaviour allows us to quantify changes in VSC that depend on subradiant versus radiant cavity resonances. Remarkably, we find evidence that the polariton states due to VSC are fully subradiant or “dark” when the cavity is fully subradiant, i.e. when probed at normal incidence, whereas the *N−1* states show radiant character. At oblique angles, when the mode has greater dipole character, the observed strong coupling behavior is comparable to the purely dipolar cavity. These findings have significant implications for modulating radiative properties of hybrid polaritons states, and outline opportunities for direct spectral characterization of the *N−1* states during polariton chemistry.

## 2 Results and discussion

### 2.1 Far-field properties of plasmonic nanorod arrays

**Figure 2:**
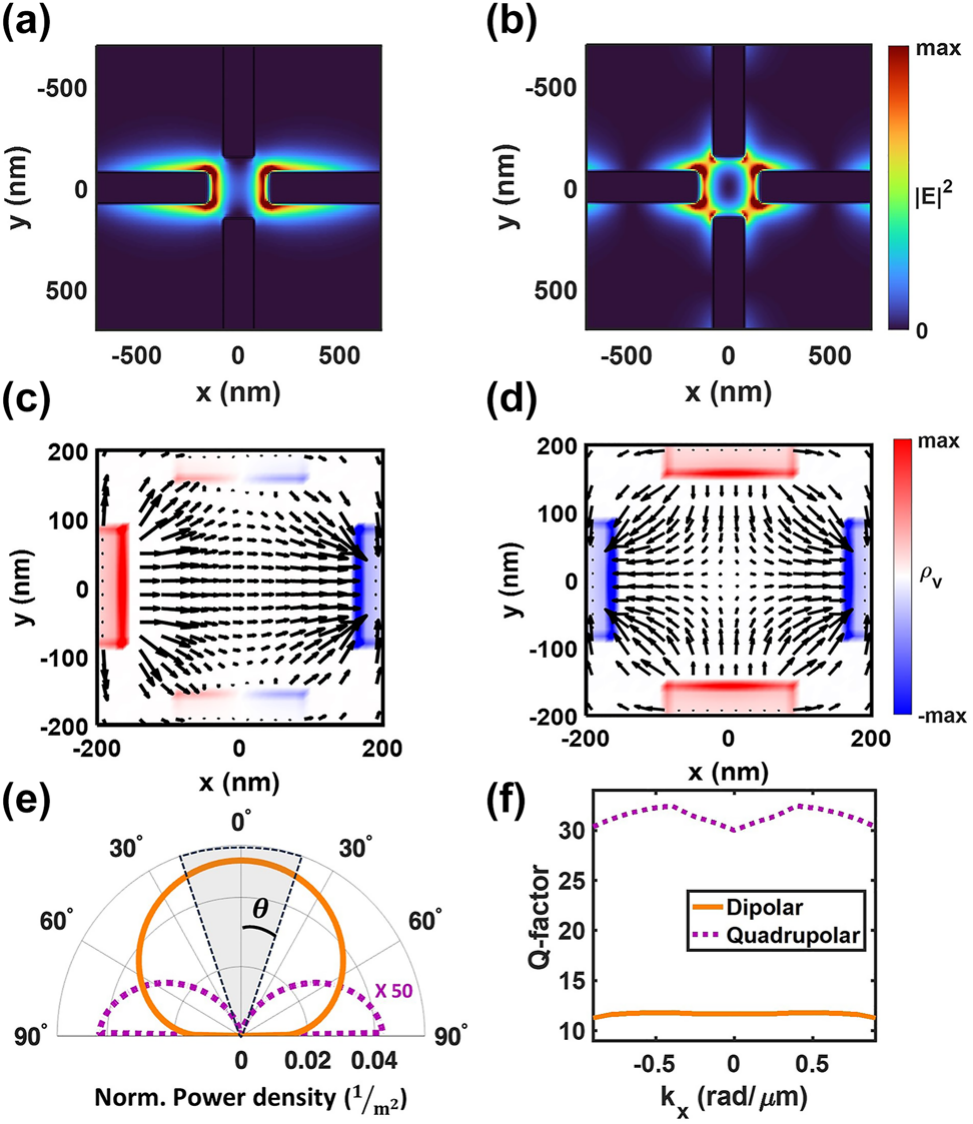
Near-field properties of the 1st order dipolar (DPL) and the 2nd order quadrupolar (QPL) mode. (a, b) Top-down view of the electric field enhancement (|*E*|^2^) at *z* = 50 nm above the SiO_2_ substrate for the (a) DPL and (b) QPL resonances. |*E*|^2^ at each location was normalized to the maximum value in the *xy*-plane. (c, d) The induced volume charge density (*ρ*_v_) and the electric field vector of the (c) DPL and (d) QPL mode in the junction region between the nanorods is plotted. Positive charge density is colored in red, and negative charge density in blue. The absolute value at each location was normalized to the maximum value in the *xy*-plane. The electric field enhancement and the induced charge density distribution correspond to the *xy*-plane at *z* = 50 nm and 100 nm above the SiO_2_ substrate, respectively. (e) Polar plot of the angle-dependent power density emitted into the far-field by the DPL (orange) and QPL (purple dotted). (f) The calculated *Q*-factor of the DPL (orange line) and QPL (purple dotted line) as a function of *k*_*x*_.

We designed a plasmonic nanostructure array with non-degenerate dipolar and quadrupolar electromagnetic field resonances in the mid-IR region, see Figures 1 and 2. The design is based on longitudinal plasmonic modes in high-aspect-ratio metal nanorods [35], [36], [37]. We arranged Au nanorods with fourfold rotational symmetry on top of a *h*_*d*_ = 40 nm SiO_2_ layer and *h*_*m*_ = 100 nm Au film. Figure 1a is a schematic of a unit cell with the electric field polarization of TM- and TE-polarized planewaves depicted. Each unit cell had a rod width, *w* = 0.15 µm, height, *h*_*m*_ = 0.1 µm, and a gap spacing of 0.3 µm × 0.3 µm (g × g), which was kept constant in all experiments. The length, *L*, of the rod was the only parameter that was modified to tune the frequency of the resonant mode. We first present the calculated absorption of planewave radiation by a plasmonic array with *L* = 1.4 µm obtained using full-wave electromagnetic simulations (finite-difference time-domain method, Lumerical Inc.; for simulation details see Supplementary Section 1.1, and Figure S1). The range of incident angles displayed is from −17.5° to 17.5° with respect to the surface normal, corresponding to the experimental angle range provided by the IR microscope objective (numerical aperture, NA = 0.3). As indicated by dash and dotted lines in Figure 1c, there are two plasmonic modes: a low energy mode around 2000 cm^−1^ and a higher energy mode near 4000 cm^−1^. These correspond to the first order and second order longitudinal resonance of the Au rods, respectively. These resonances show distinct absorption spectra as a function of incident angle, *θ*, and polarization in agreement with studies of single metal nanowires [37], [38]. We also show the dependence on the in-plane Bloch wavevector, with *k* = *k*_*x*_ or *k*_*y*_ equivalently based on symmetry (SI Section 1.2 for simulation details). The 1st order mode maintained high, resonant absorption near ∼70 % with no angle-dependent frequency dispersion for TM- or TE-polarized incident light. The 1st order mode is thus radiant or “bright” regardless of angle. In contrast, the 2nd order mode is completely subradiant or “dark” at *θ* = 0°, with the absorption of planewave radiation increasing as *θ* increases. This absorption into the 2^nd^ order mode is only possible with TM-polarization, because there is a non-zero *E*_*z*_ component with some retardation along the length of the rod’s long axis, breaking the mode symmetry. These simulations highlight that the 2nd order mode has a non-zero dipole moment as *k*_*x*_ increases, allowing it to be probed spectroscopically with planewave excitation at oblique angles. We note that this modal behavior resembles so-called “bound states in the continuum” (BIC) recently being studied in a variety metamaterials [39], [40], [41] (see Figure S1 for the full angle-dependent dispersion). We believe this study is the first showing how BIC modes impact VSC. The experimental spectra (Figure 1d) collected at normal incidence on arrays with different nanorod lengths (*L*) show that both plasmonic modes are apparent. The higher energy 2nd order mode is weakly resolved, despite being completely subradiant at normal incidence, due to the finite angular range of the IR microscope numerical aperture.

### 2.2 Near-field properties of plasmonic nanorod arrays

We first computationally analysed the local electric field profile of nanorod arrays that are excited by TM-polarized planewaves at *θ* = 0° and 17.5° in order to probe the 1st order and 2nd order modes, respectively (Figure S2). The electric field profile of the 2nd order mode clearly shows quadrupole field distributions at the center of the unit cell, albeit weakly and with some asymmetry. The weak excitation of the quadrupole is due to retardation along the nanorods during planewave illumination, as discussed above. For an electromagnetic mode with subradiant behaviour, such as a BIC, it has been shown that the local electrical field distribution can be probed computationally using a fictious local source that matches the mode symmetry [41]. Therefore, to better understand the near-field properties of the ideal subradiant resonance supported by the nanorod array, we used a pseudo-electric quadrupole (PEQ) source. Two dipole point sources oriented along the *x*-axis were located at the centre of the unit cell. These sources were separated in space by 20 nm and were 180° out of phase with one another. This setup generates a subradiant quadrupole source that exchanges IR energy only via near-field interactions, i.e. radiation into the far-field is forbidden by symmetry (more details in SI Section 1.3). In comparison, the near-field properties of the 1st order mode are readily resolved by excitation with a single electric dipole (ED) source oriented along the *x*-axis at the center of the unit cell. Figure 2a and b shows the electric near-field enhancement profile at the frequency of 1st order and 2nd order mode on an *L* = 1.4 µm array using the ED and PEQ source, respectively. Due to the four-fold symmetry of the rods, the 1st order dipole resonance was only induced along the direction of the electric field polarization of the ED source. However, the PEQ source clearly excited the quadrupolar field distribution of the 2nd order resonance at the junction between all four nanorods. We next calculated the distribution of induced charge density in the center of the unit cell and compared this to the electric field vector map. The plotted data corresponds to an instant during the optical cycle at 90° phase, when the polarization is most pronounced (Figure 2c and d). For the 1st order mode, intense positive charge density (red) and negative charge density (blue) is induced on the left and right edges of the horizontal nanorods, as expected for a dipolar resonance. Only small regions on the edges of the perpendicular nanorods show induced charge density, so most of the electric field points from left to right, giving rise to a strong net dipole moment. For the 2nd order mode, positive and negative charge is induced with even symmetry over the *xz* and *yz* mirror planes, which is expected for quadrupolar resonances [42], [43], [44]. Therefore, this mode has no net dipole moment, and is entirely subradiant at normal incidence. However, the symmetry of the induced mode is easily broken by a non-normal planewave with TM-polarization as confirmed in Figure 1c and d.

The differing symmetry of the resonances also results in distinct far-field radiation patterns, as shown in Figure 2e. The radiation from the dipolar resonance (orange) shows the conventional cos(*θ*) profile associated with dipole radiators, while the quadrupolar resonance (purple dotted) only radiates at high angles. In addition, the normalized power density emitted by the quadrupolar resonance was much less, so it is multiplied by a factor of 50× in the visualization. The calculated *Q*-factor (Figure 2f) also indicates more efficient electromagnetic energy concentration by the quadrupolar resonance compared to the dipolar resonance. Over the range of Bloch vectors, the *Q*-factor of the quadrupolar resonance is more than double that of the dipolar resonance. Although the *Q*-factor of quadrupolar resonance is lower than what has been reported recently for other BIC modes [39], [41], [45], the enhancement is quite significant for a BIC in a relatively lossy plasmonic structure. Here the *Q*-factor was estimated as *Q* = *ω*/Δ*ω*, where Δ*ω* is the full-width at half-maximum. For more simulation details relating to the far-field projection and *Q*-factor analysis, see SI Section 1.4–5. These results again confirm the distinct behavior resulting from the dipolar or quadrupolar mode symmetry of the plasmon resonances, termed “DPL” or “QPL” respectively, throughout the rest of this report.

### 2.3 Vibrational strong coupling to the DPL and QPL modes

**Figure 3:**
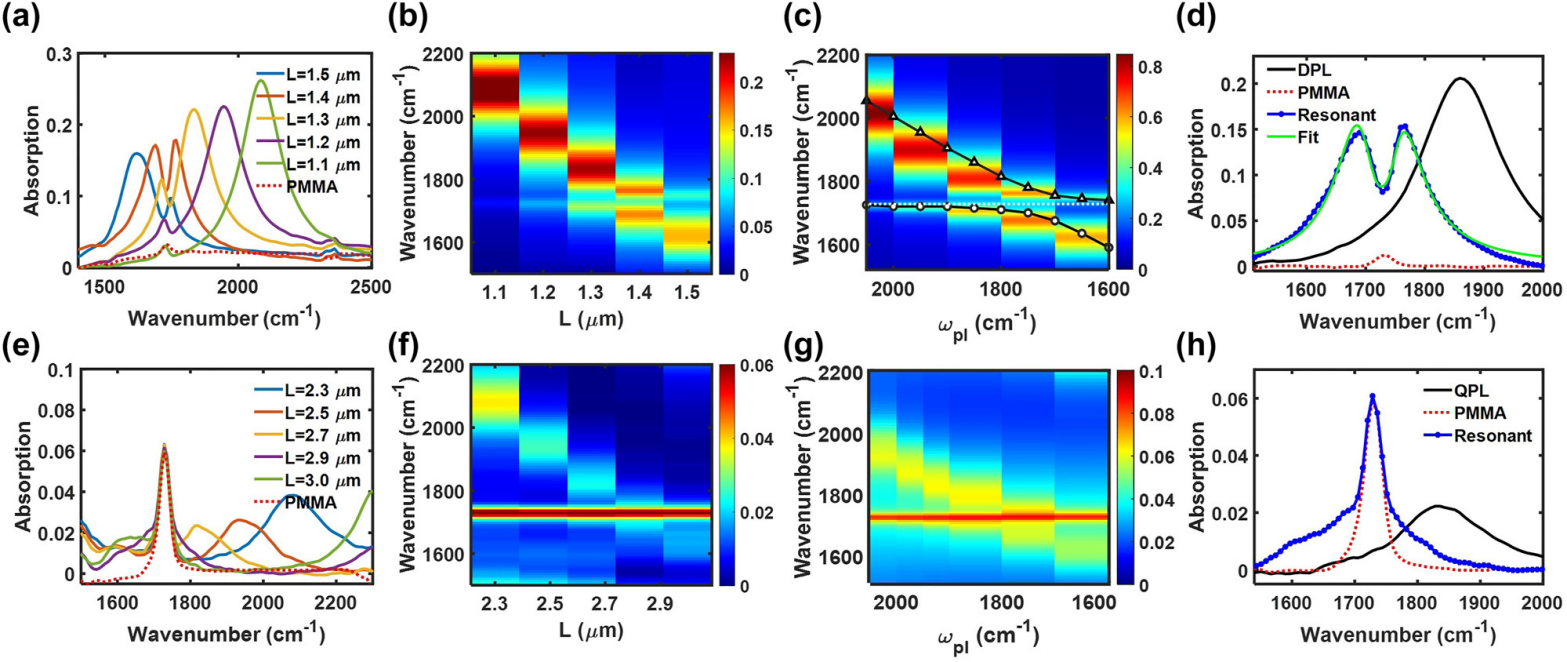
Far-field VSC behavior. (a, b) Experimental absorption measured when dipolar plasmons (DPL) were tuned through the C=O stretching mode of PMMA. The red dotted trace is a control PMMA spectrum. (e, f) Experimental absorption measured when quadrupolar plasmons (QPL) were tuned to the molecular vibration frequency. (c, g) Simulated dispersion reproducing (b, f). In simulation, the quadrupolar resonances were excited off-axis (*θ* = 17.5°). The fitted dispersion of the upper (triangle) and lower (circle) polariton branches and the uncoupled molecular vibration (white dotted) are also plotted on (c). (d, h) Experimental spectra for resonant cases (blue dots). For the DPL-molecular system, the experimental spectrum was fit to the two-coupled oscillator model (green). The substrate before deposition of PMMA (black), and the control PMMA spectrum (red dash) are also plotted.

We investigated vibrational strong coupling between the DPL or QPL mode and the carbonyl stretch (C=O) of polymethyl methacrylate (PMMA) thin films, near the infrared frequency 1729 cm^−1^. It is well established that the C=O stretch of PMMA allows for collective VSC with a variety of radiant cavity modes, e.g. Fabry–Pérot cavities [46], [47] and plasmonic cavities [48], [49], [50]. Here we additionally probe the interaction with a subradiant cavity resonance. We can probe this behaviour using far-field IR spectroscopy due to the BIC-like modal behavior that transitions between subradiant and radiant based on incident angle. Figure 3a, b, e and f shows the experimental IR spectra when the DPL and QPL are tuned through the C=O stretch, respectively. Clear anti-crossing behavior and a dip in absorption at the molecular vibrational energy (1729 cm^−1^) are observed for the DPL case in Figure 3a and b as expected for VSC. However, when the weak far-field signal associated with the QPL mode is tuned across the molecular absorption (Figure 3e and f) neither the anti-crossing signal nor an absorption dip is apparent. Numerical simulation (Figure 3c and g) of absorption upon planewave excitation showed excellent agreement with experiment, although there is overall decrease in absorption intensity experimentally compared to simulation likely due to imperfections in the fabricated samples. Note the off-axis excitation provided by the numerical aperture of the FTIR objective gave rise to the weak QPL signal observable in the far-field. To account for this effect in calculations, the simulated QPL arrays in Figure 3g were excited at an angle of incidence *θ* = 17.5°. The excellent experimental agreement with the computational results lends confidence that the simulations may be used to support further analysis.

By fitting the on-resonance spectrum of the coupled DPL-molecular system to a classical two-coupled oscillator model (see SI Section 2.3), we obtained the collective coupling strength *g*_*N*_ = 73 cm^−1^. As pointed out by Leng et al. [18], *g*_*N*_ from our fitting is equal to the on-resonant vacuum Rabi frequency, which differs by a factor of 2 from the definition of *g*_*N*_ in some cases in the literature [30]. Therefore, $g_{N}=2\sqrt{N}g_{s}$ , where *g*_*S*_ is the coupling strength of a single molecule with one cavity mode. The fitted value of *g*_*N*_ for the DPL system was at the onset threshold for strong coupling, defined as $g_{N}\geq \gamma_{vm}/\;,2+\gamma_{\mathrm{dpl}}/\;2$ [30], [51], [52]. The fitted values for *γ*_*vm*_ and *γ*_*dpl*_ were 25 cm^−1^ and 119 cm^−1^, and they correspond to the damping rates of the C=O stretch and dipolar plasmon resonance, respectively. These extracted fit values were used to plot a theoretical dispersion of the upper and lower polariton branches in Figure 3c, which shows excellent overlap with both the numerical simulation as well as the experimental spectra. In contrast, the on-resonant spectra of the QPL-molecular system (Figure 3h) gave a poor fit to the two-coupled oscillator equation. At first glance, it may seem that the on-resonance spectrum (blue trace, Figure 3h) could simply be the result of the summed spectral counts of the control PMMA absorption spectrum (red dash) and the QPL resonance that is red-shifted due to the refractive index change induced by PMMA, without evidence for strong coupling. However, the formation of polaritons on the QPL substrate is supported by further computational analysis of the angle-dependent behavior of the coupled system in the limiting cases when the QPL mode has maximum dipolar character, versus when the QPL is entirely subradiant, as well as experimental quantification of the absorption cross-sections of the resulting spectral signals.

### 2.4 Computational analysis of far-field and near-field coupling to the QPL

**Figure 4:**
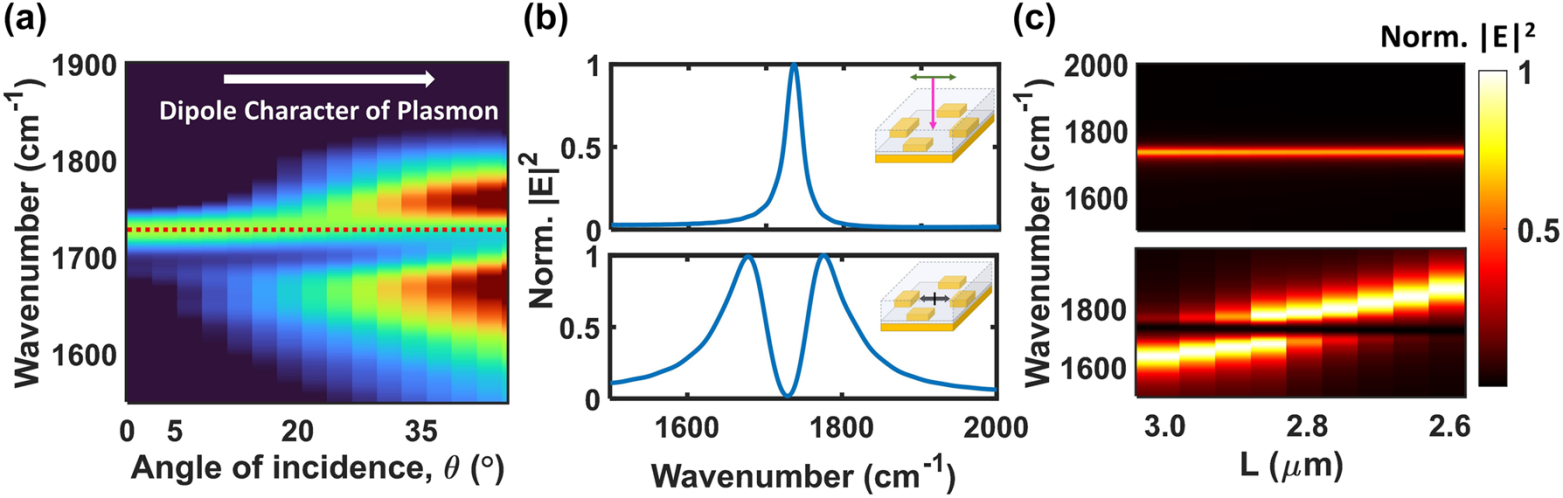
Analysis of the QPL-PMMA coupled system. (a) The calculated field enhancement spectrum as a function of in-plane wavevector or angle of incidence, *θ* (°), i.e. the far-field angle dispersion. The QPL resonance is at the same frequency as the C=O stretch of PMMA (red dotted). (b) Calculated field enhancement spectrum for the same QPL-PMMA system when excited by far-field planewave at normal incidence (upper panel) or when excited by a PEQ source in the near-field (lower panel). (c) Simulated frequency dispersion as a function of the QPL resonance based on nanorod length, *L*. The upper panel shows the response to planewave excitation at normal incidence. The lower panel shows anti-crossing of the polariton branches that is measured only within the near-field when excited by a PEQ source.

The QPL cavity resonance gains dipolar character at higher angle during planewave excitation, i.e. with higher in-plane wavevector, as in Figure 1c. Therefore, we investigated if the greater dipole character of the cavity mode allows for the observation of polariton states in the far-field, indicating that strong coupling has been achieved. In simulation (Figure 4), we excited the QPL-PMMA system when the QPL is tuned to the C=O stretching frequency. The simulation used an electric dipole source (ED) and the Bloch wavevector was swept from *k*(0, 0) to *k*(0.5, 0). This setup is equivalent to planewave excitation with non-zero angle of incidence, *θ*, thus analysis in terms of wavevector was converted to *θ* for easier interpretation. The eigenmodes of the system were calculated by performing a Fourier-transform of the time-decaying electric field recorded around the gap region. The results shown are limited to a maximum angle of *θ* = 40°, because higher angle spectra show interference with other diffraction modes that are irrelevant to the discussion in this section. The data in Figure 4a shows how the spectrum transitioned from a single peak at the C=O vibrational frequency into two distinct polariton peaks as *θ* increases. The perfect quadrupolar symmetry is broken at higher angles, giving rise to dipolar mode character. Only at higher angles do the hybridized polariton states take on the radiant, dipole character of the cavity allowing the upper and lower polariton signal to be observed in the far-field. Furthermore, as *θ* increases the molecular absorption at 1729 cm^−1^ also decreases. The decreasing molecular absorption is expected as the dipolar character of cavity mode increases, thereby promoting the *N−1* molecule-like states to become more “dark” or subradiant in character. In the limit that the upper and lower polariton gain a strong dipole moment, the summed dipole moments of the rest of the *N−1* states cancel out, which is the well-established result from classical and quantum mechanical models of collective strong coupling [11], [12], [53], [54]. This behavior is in stark contrast with the lack of angle-dependent trends when the DPL mode is tuned to the molecular frequency (see SI Figure S3).

Now, consider when *k*_*x*_ = 0 or *θ* = 0°, when the quadrupolar resonance cannot be excited by plane wave excitation, and only molecular absorption at the unperturbed C=O vibrational frequency is observed. Because of the quadrupolar symmetry of the QPL, strong coupling between the QPL mode and the dipole moments of C=O stretches in the PMMA film is only possible via near-field energy transfer. These “dark” polaritons are readily observed when simulating near-field spectra. Clear Rabi splitting is apparent in the lower panel of Figure 4b, corresponding to the calculated near-field spectrum when the resonant QPL-PMMA system is excited by a PEQ source in the gap region, with *k*_*x*_ = 0. A collective coupling strength of *g*_*N*_ = 103 cm^−1^ is extracted from the fit. This value is larger than the experimental Rabi splitting measured for the resonant DPL case (Figure 3d, *g*_*N*_ = 73 cm^−1^) and is well above the threshold for VSC. We hypothesize that the numerical simulation predicts higher coupling strength for the QPL compared to the DPL due to the higher *Q*-factor (*γ*_*qpl*_ = 107 cm^−1^, extracted from the simulation). In comparison, the upper panel of Figure 4b shows the spectral response of the gap region of the QPL when the system is excited by an ED source. This data once again confirms that the only signal that can be observed in the far-field is at the same frequency as the unperturbed molecular vibration, even though coupling via near-field interactions is expected to promote strong coupling and hybridized polariton states. Finally, we also confirmed the anti-crossing behavior of the subradiant polaritons as a function of the QPL cavity resonance dispersion (Figure 4c, lower panel) probed by varying the nanorod length. The anti-crossing is only manifest within the near-field of the geometry, whereas the calculated signal that can be probed in the far-field is shown in the upper panel in Figure 4c.

**Figure 5:**
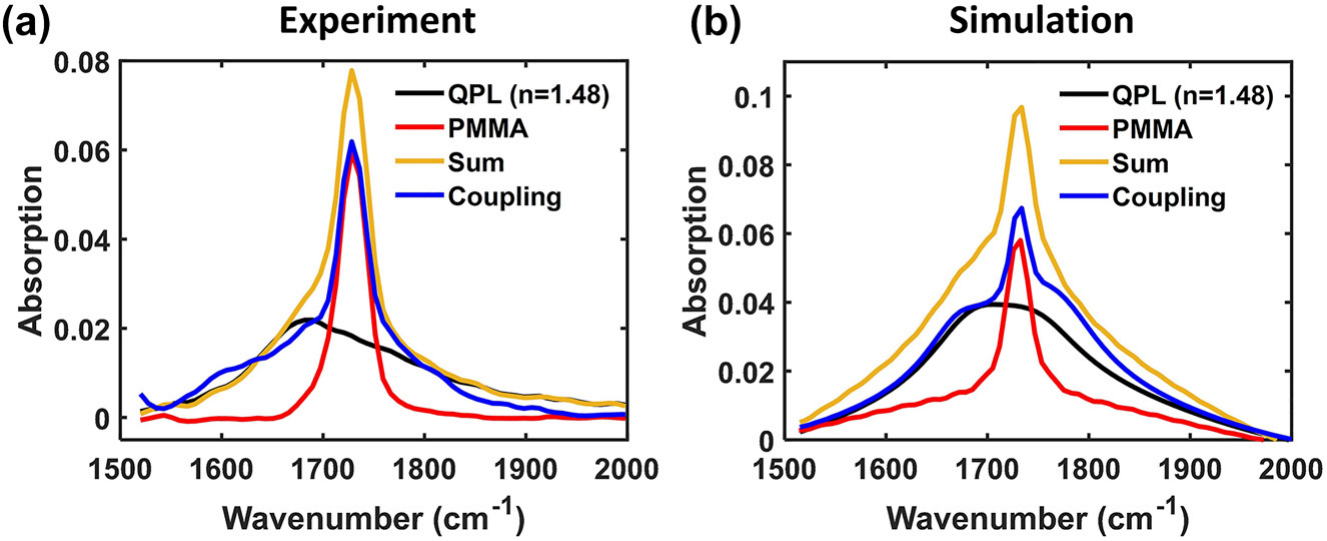
Far-field spectral analysis of the QPL-PMMA coupled system. (a) Experimental absorption of PMMA (red) and the bare QPL mode (black). The QPL spectrum is produced by red-shifting and broadening the measured, bare QPL spectrum based on the refractive index of PMMA. See text for details. The numeric sum of these signals (yellow) is compared to the measured coupling spectra (blue) after PMMA deposition on the QPL array. (b) Calculated absorption of the same QPL-PMMA system when excited from the far-field with a planewave at *θ* = 17.5°.

Our simulation results in Figure 4a–c indicate that, even though it is unambiguous that the system is strongly coupled within the near-field, the far-field signal looks very similar to uncoupled molecules. However, if there were no coupling between the QPL and the C=O stretching mode in the experimental FTIR spectrum (Figure 3h), we would expect greater absorption, corresponding to the numeric sum of the two spectral components of the isolated cavity and isolated molecular film. A fictitious spectrum corresponding to this sum of the spectral components is shown with a yellow line in Figure 5a. Importantly, the experimental coupling spectra in blue exhibits significantly lower absorption. We interpret this lower absorption as key evidence of VSC between the QPL mode and PMMA that can be observed in the far-field spectrum. Effectively, the off-axis excitation provided by the numerical aperture of the microscope allowed us to probe some of the decrease in counts associated with the *N−1* states as the cavity gains more dipolar “bright” character, as shown in the angle-dependent far-field spectra associated with VSC in Figure 4a. Our experimental trend is also reproduced qualitatively in the simulated absorption spectra shown in Figure 5b. The black line corresponds to the QPL mode embedded in a planar film with the same refractive index as PMMA (*n* = 1.48), but without absorption in the dielectric function. The simulated excitation is a planewave at *θ* = 17.5°. Note that the experimental QPL mode (black trace, Figure 5a) was generated by red-shifting and broadening the measured plasmon mode (black trace, Figure 3h) according to the computational result before and after introducing the *n* = 1.48 film. Furthermore, it appears that the slight broadening in the experimental coupling spectra (blue trace) may be another line of evidence for VSC, indicating the onset of bright character for the upper and lower polariton states, though the effect is more obvious in simulation. More analysis of the broadening can be found in SI Figure S4. Thus, it is plausible that the experimental spectra indicate “dark” polaritons due to VSC between the QPL mode and PMMA in the near-field, even though it is the decrease in intensity of the *N−1* states associated with the off-axis, bright character of the polaritons that supports this conclusion. These trends are an important insight into how strong coupling can be monitored in resonant geometries that support subradiant modes, and our data is consistent with theoretical predictions [33], [34] and corroborates recent experimental observations of Rabi splitting in subradiant cavities using EELS [31] and s-SNOM [32]. Future studies can use these alternative characterization techniques to directly probe the high quality, “dark” vibrational polaritons supported by the QPL modes discussed here.

**Figure 6:**
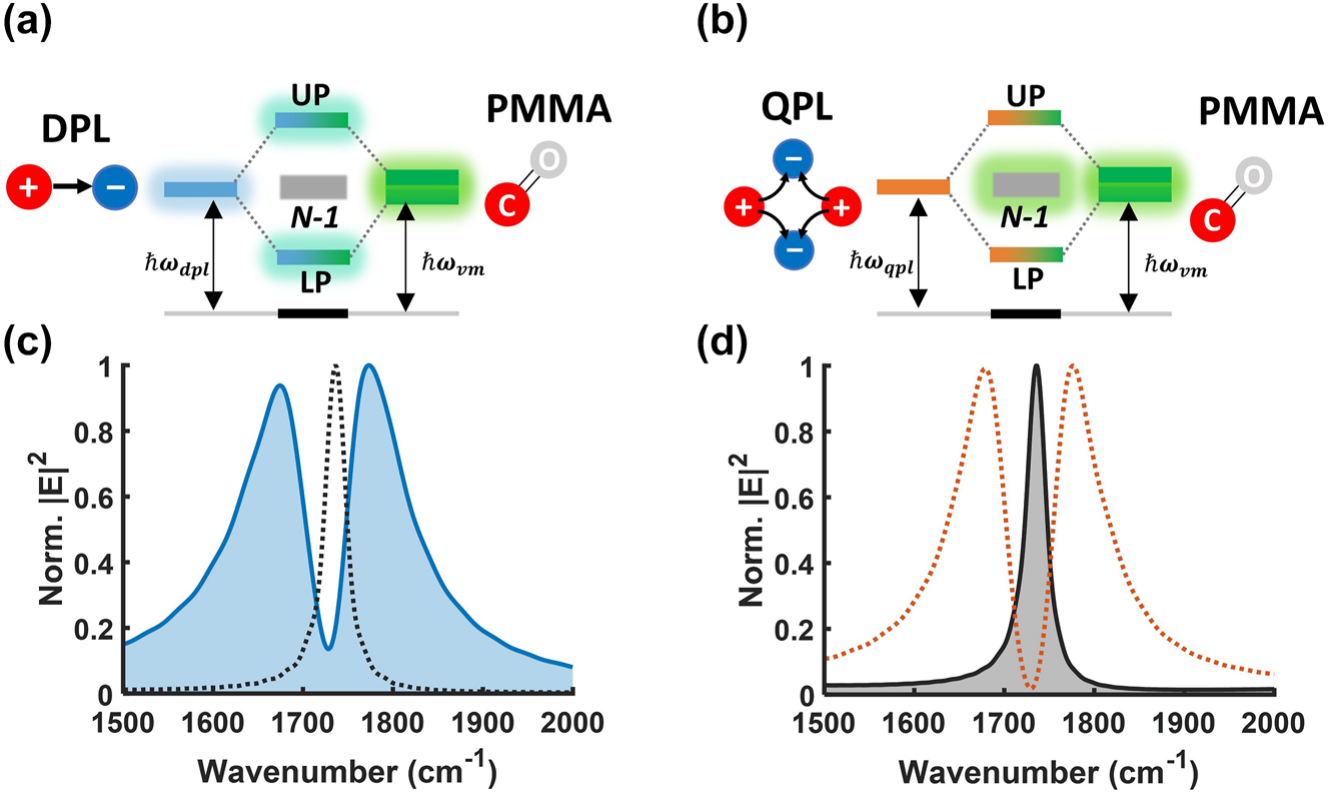
Energy diagram of the coupled system. (a) Collective VSC between the DPL mode and PMMA results in two “radiant” polaritons (UP, LP) and *N−1* “subradiant” states at the unperturbed molecular frequency. (b) The QPL-PMMA counterpart results in two “subradiant” polaritons (UP, LP) and *N−1* “radiant” states. States with radiant character are illustrated with glowing edges. (c, d) Normalized electric field enhancement spectra of the DPL-PMMA or QPL-PMMA system calculated by exciting either with an ED (net-dipole moment, filled spectra) or PEQ (zero-dipole moment, unfilled spectra). (c) For the DPL-PMMA system, the two polariton states are observed after excitation by an ED source, indicating that they take on the radiant character of the DPL mode. The resulting UP and LP states are plotted in the shaded blue spectrum. On the contrary, the *N−1* molecular states have subradiant character and can only be excited by a PEQ source, as shown in the grey dotted spectrum. (d) For the QPL-PMMA system, the *N−1* molecular states have been excited by an ED source, indicating their radiant character, and they are plotted in the filled grey spectrum. The polariton states are excited by a PEQ source, indicating their subradiant character. The UP and LP of the QPL-PMMA system are plotted in the unfilled orange spectrum.

A final insight from our study relates to the radiant or subradiant character of the *N−1* molecule-like states that result from collective VSC. Because our data strongly suggest that the QPL resonance and the molecular ensemble are strongly coupled, regardless of the far-field observation of the polariton states, we also expect the presence of *N−1* states at nearly the same energy as the unperturbed molecules. According to the classical coupled oscillator model, where *x*_*cav,dpl*_ is the displacement of a dipolar cavity oscillator, when *N* molecules couple to one radiant or “bright” cavity mode with non-zero dipole moment (i.e. $x_{\mathrm{cav,dpl}}\left(t\right)\neq 0$ ), there are 2 polariton states with radiant character and there are *N−1* subradiant molecule-like “dark” states with a net displacement that sums to 0 [12]. The quantum mechanical Tavis–Cummings model also predicts that the *N−1* molecule-like states are collective “dark” states with transition dipoles cancelling to zero, indicating that they exhibit little to no character of the radiant dipolar cavity mode [54]. On the contrary, when VSC occurs in cavity mode with a net-zero dipole moment ( $x_{\mathrm{cav,qpl}}\left(t\right)=0$ ), such as the subradiant QPL cavity discussed above, the 2 polariton states take on the symmetry of the cavity resonance, and are thus subradiant as well [33], [34]. However, *N−1* states are still expected to adopt a symmetry that cannot exchange energy with the quadrupolar cavity oscillator. Therefore, in this case, the *N−1* states gain dipolar character, and correspond to collective radiant or “bright” states delocalized across the *N* molecules in the ensemble. This conversion between the radiant and subradiant character of the polaritons and the *N−1* molecule-like states depending on the symmetry of the cavity resonance is depicted in schematic in Figure 6a and b. States with radiant character are illustrated with glowing edges. Below each figure the simulated electric field spectra obtained using an either ED or PEQ source are also displayed. The spectra are normalized to their maximum so that the Rabi splitting and the spectral signal corresponding to the *N−1* states can be compared more easily. When the DPL-PMMA system is excited by an ED, the electric field spectra show clear polariton peaks (Figure 6c, blue solid), but the QPL-PMMA counterpart showed only a single peak at the same energy as the unperturbed molecular absorption (Figure 6d, grey solid). Conversely, when both systems are excited by a PEQ source having zero dipole moment, the near-field enhancement spectra show opposite behavior. That is, the *N−1* states appear in the DPL-PMMA spectrum (Figure 6c, grey dotted), while polariton states are observed in the QPL spectrum (Figure 6d, orange dotted). Therefore, we interpret our experimental spectra in Figure 3h as evidence for *N−1* radiant states as a result of collective VSC between the QPL substrate and C=O stretches in PMMA, with a small loss of counts due to the bright character of the cavity when there is some off-axis excitation.

We acknowledge that there is still a significant population of uncoupled molecules on the substrate for both the QPL-molecular system and the DPL-molecular system. We estimated the ratio of coupled C=O bonds to the total C=O bonds that were deposited by comparing the experimentally obtained *g*_*N*_ with the theoretical single molecule *g*_*S*_ for DPL-PMMA system and the molecular density of the PMMA film. In fact, by this estimate, the coupled molecules accounted for only ∼6 × 10^−3^ % of estimated total number of C=O molecules in a unit cell of the cavity. Nonetheless, the resonant spectra of DPL-PMMA coupled system (Figure 3d) show little contribution from these uncoupled molecules in the fitted absorption spectra. Therefore, for the QPL-PMMA coupled system that displays an even stronger coupling strength in the near-field, it is reasonable that the uncoupled molecules have little contribution to the total molecular absorption measured as well. More details on the estimation of the number of deposited and coupled C=O bond on the DPL or QPL-PMMA system can be found in SI Section 2.4.

## 3 Conclusions

We have developed nanophotonic Au substrates that support IR quadrupolar plasmonic resonances (QPLs) to study VSC. This QPL cavity design is subradiant or “dark” at normal incidence and acquires radiant, dipolar character as the in-plane wavevector is increased. A thin film of PMMA was deposited on the QPL cavities to promote VSC with the C=O stretch in PMMA. Experimentally, the absorption spectra of the QPL-PMMA system show little evidence of Rabi splitting or anti-crossing at normal incidence. However, numerical analysis indicates the existence of “dark” polariton states in the near-field and “bright” *N−1* collective molecular states that can be probed with far-field spectroscopy. This result aligns well with classical and quantum mechanical VSC models that predict hybrid polariton states with cavity-like radiative character, and *N−1* collective molecular states with little cavity character. We interpret our experimental data as confirmation that subradiant cavities promote subradiant hybrid polariton states that are limited to within the near-field of the cavity mode, while the *N−1* collective molecular states are the dominant contribution observed in the experimental absorption spectra. We anticipate that cavity designs that allow for direct experimental observation of the *N−1* collective molecular states may aid further studies of polariton chemistry aimed at understanding the role of these *N−1* states.

## Supplementary Material

Supplementary Material Details
