# Supplementary material for: Subradiant plasmonic cavities make bright polariton states dark
Source: Nanophotonics. 2024 Mar 22;13(11):2035–45. doi: 10.1515/nanoph-2024-0058 (PMC11501913; doi:10.1515/nanoph-2024-0058)
Supplement: Supplementary file 1 — Supplementary Material Details [file j_nanoph-2024-0058_suppl_001.docx]

**Supporting Information for**

**Subradiant plasmonic cavities make bright polariton states dark**

**Ju Eun Yim, Zachary T. Brawley, and Matthew Sheldon***

**Table of Contents**

Section 1. FDTD Simulation

- 1. General simulation setup & calculation of planewave absorption
  2. Calculation of mode dispersion diagram
  3. Near-field simulation
  4. Near-field to far-field transformation
  5. Q-factor calculation
  6. Cavity mode volume calculation

Section 2. Experimental Setup

2.1. Fabrication

2.2. FTIR measurement

2.3. Two-coupled oscillator model

2.4. Estimation to the number of C=O bonds in a single unit cell

Section 3. References

1. **FDTD Simulation**
   1. **General simulation setup & calculation of planewave absorption**

We performed our full-wave electrodynamic simulation using the finite-difference time-domain method (Lumerical, Ansys Inc.). Plasmonic nanocavity consisted of a gold nanorod with width, **w** = 0.15 $\mu$m, height, **h** = 0.1 $\mu$m, and a gap spacing of 0.3 $\mu$m X 0.3 $\mu$m (**g** X **g**), which was kept constant in all simulations. The length, **L**, was the only parameter that was modified to tune the frequency of the resonant modes. Below the nanorods, is a 40 nm of SiO_2_ layer and 100 nm of Au film to create metal-insulator-metal structure. An infinite plasmonic nanorod array was simulated adapting periodic boundary conditions (BC) in x- and y-boundaries of a unit cell shown in the lower part of **Figure 1a** in the main context. The z min and z max boundaries were set to perfectly matched layer BCs. The permittivity values used for the simulation was taken from the Palik [1] for Au and SiO_2_, and from Zhang *et al*. [2] for PMMA.

The planewave source bandwidth was 104 THz, simulating broadband planewave with a wavelength range of 2 ~ 6.6$\mu m$. For non-normal incidences, Broadband Fixed Angle Source Technique (BFAST) was used to ensure no angular dispersion of illumination depending on the frequency [3]. The TM- and TE-polarized planewaves were modelled by aligning the electric-field polarization parallel and perpendicular to the plane of incidence (xz-plane), respectively. The far-field absorption by a plasmonic array was calculated by subtracting percent reflection and transmission from unity, which were measured above the planewave source and 100 nm below the bottom gold film.

When calculating the absorption as a function of incident angle, we confined the maximum angle to be $\boldsymbol{\theta}_{\boldsymbol{max}}$ = 17.5°, corresponding to the experimental angle range provided by the IR microscope objective (15X Cassegrain objective, NA = 0.3). We acknowledge that due to the geometry of reflective objective, perfect normal incidence ($\boldsymbol{\theta}$= 0°) is not possible. However, we studied ideal modal behavior at normal incidence as well.

- 1. **Calculation of mode dispersion diagram**

To only excite plasmonic modes of interest corresponding to TM- or TE-polarized plane wave excitation, one electric dipole (ED) source was placed at the center of the unit cell, and it was x- or y- oriented, respectively. The frequency range of the dipole source was set to cover 45 ~ 150 THz. Thirteen random time-monitors were located close to the structure. Start apodization was applied and set to record electric field in time ($E_{x}\left( t \right), E_{y}\left( t \right),$and$E_{z}(t)$) from 70 fs to remove the source signal. The zero padding of 2^16^ was used for Fast-Fourier Transform (FFT) to increase the frequency resolution. The resultant Electric field intensity as a function of frequencies ($\left| E(\omega) \right|^{2}$) was then normalized to the Fourier-Transformed (FT) spectrum of the sources and averaged over all the time-monitors used in the simulation. Bloch boundary conditions were used in x and y boundaries, and PML was used in z boundary. To calculate the frequency of plasmonic modes as a function of spatial in-plane wavevector, a sweep over **k** = (**k_x_**, **k_y_**) value from **k**($\Gamma$) = (0,0)($\frac{2\pi}{P}$) to **k**($X$) = (0.5,0)($\frac{2\pi}{P}$) was performed, where $P$ is the periodicity of the array. The same FFT calculation was done after every different **k_x_** simulation. The maximum **k_x_** value shown in **Figure 1c** was calculated from the equation below.

$$k_{x, max}=\frac{2\pi}{\lambda_{0}}sin(\boldsymbol{\theta}_{\boldsymbol{max}})$$

where $\lambda_{0}^{-1}$ is the wavenumber and the maximum shown in the y axis was 4,000 cm^-1^. $\boldsymbol{\theta}_{\boldsymbol{max}}=$17.5$^{\circ}$ was used as mentioned above. The top x-axis of corresponding $\boldsymbol{\theta}$ was calculated with the above equation as well.


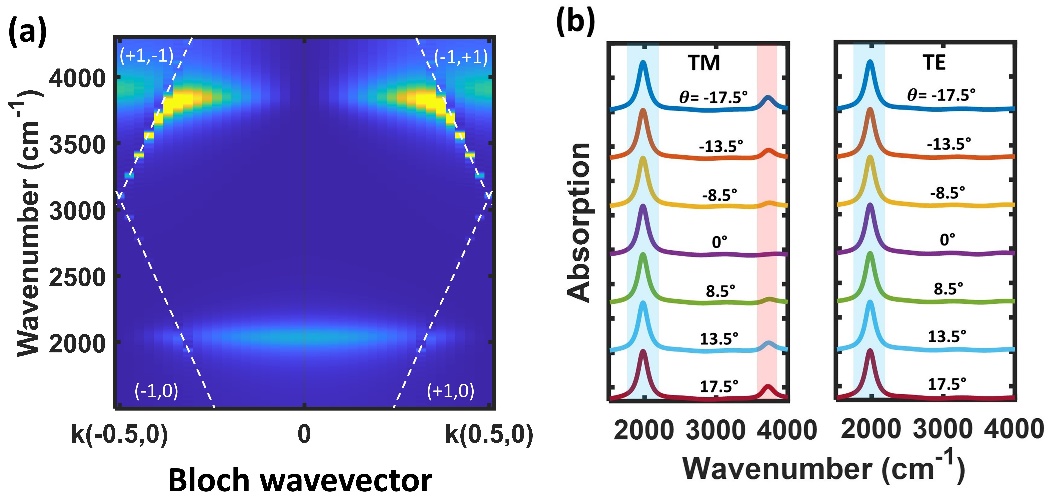


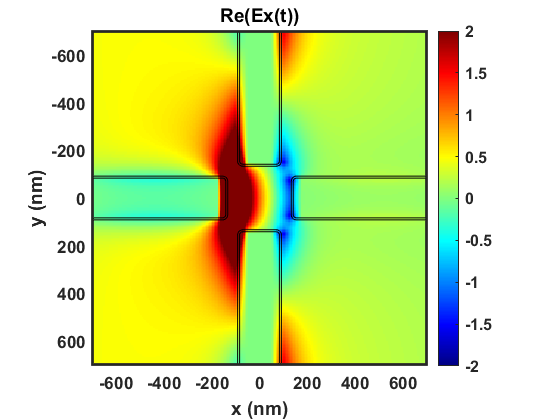

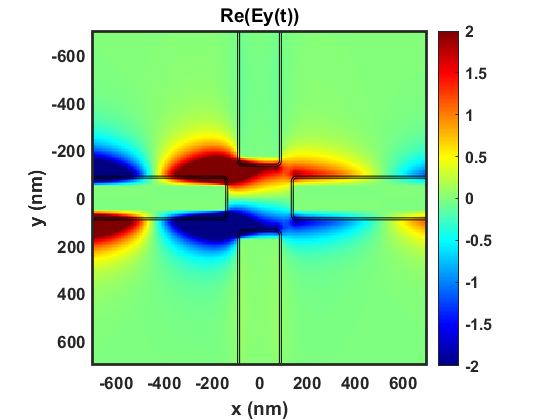


**Fig. S2** Real part of Electric field taken from the 1^st^ order (at 1,972 cm^-1^) and 2^nd^ order resonance (at 3,717 cm^-1^) induced by TM-polarized planewave. **(a, b)** 1^st^ order resonance was excited by $\boldsymbol{\theta}$= 0° and **(c, d)** 2^nd^ order resonance was excited by $\boldsymbol{\theta}$= 17.5° on an **L** = 1.4 $\mu$m array. Quadrupole field distribution at the centre of the unit cell is clear on the 2^nd^ order mode, albeit weakly and with some asymmetry. All electric field was calculated at its maximum (phase = $\frac{\pi}{2}$) during the optical cycle at the **z** = 50 nm from the SiO_2_ substrate.

**(a)**

**(b)**

**(c)**

**(d)**

**Fig. S1** (a) Full angle-dispersion diagram of plasmonic modes on an array with **L** = 1.4 $\mu$m calculated with x-oriented local dipole source. The intensity of the plot represents electric field intensity of plasmonic modes as a function of in-plane wavevector. Denoted in dotted lines are diffraction orders of the periodic array. (b) Simulated absorption of TM- and TE-polarized planewave by the same array for comparison.

- 1. **Near-field simulation**

An electric dipole (ED) was oriented along x axis at the center of the unit cell with zero Bloch wavevectors simulated far-field planewave illumination at normal incidence. We used a fictitious “pseudo-electric quadrupole” (PEQ) to excite ideal, subradiant quadrupolar resonance in the near-field. The PEQ was generated by two closely located dipoles oriented along the x axis. They were located at the center of the unit cell and were separated in space by 20 nm. One of the dipoles had 180 degrees phase shift. This setup generated quadrupolar radiation in the IR frequency region. Simulation unit cell for the calculation had Bloch boundaries with zero in-plane wavevector in x and y to mimic normal incidence. The ED and PEQ excited a nanorod array of **L** = 1.4 $\mu$m. The electric field profile ($\left| E \right|^{2}$) data was calculated at $\lambda$ = 5.51 $\mu$m for the dipolar resonance, and $\lambda$ = 2.98 $\mu$m for the quadrupolar resonance on the xy-plane at z = 50 nm above the SiO_2_. Calculation of the induced charge density distribution was done at z = 100 nm above the SiO_2_ substrate due to a much stronger electric field at the corner of the rods. We treated metal as a cloud of free electrons, so the volume charge density ($\boldsymbol{\rho}$**_v_**) was calculated by the divergence of $\varepsilon_{0}\boldsymbol{E}$, where $\varepsilon_{0}$is the free space permittivity and $\boldsymbol{E}$ is the 3D matrix of un-interpolated complex vector field as a function of position. The electric field vector map that is overlapped on top corresponds to the x- and y-component of real($\boldsymbol{E}$). We believe that slight mismatch between the charge density map and the electric field vector is induced by the $E_{z}$ component that is only included in the charge density calculation. For near-field electric field spectra shown in the manuscript **Figure 4,5**, we performed the same calculation as described in the Section 1.2 but either with ED or PEQ on the DPL- or QPL- resonant arrays.

- 1. **Near-field to far-field transformation**

Frequency-domain field and power monitor was placed 150 nm above the gold rods. This location ensures that a homogenous near-field to far-field transformation above the structure. The electric and magnetic near field data are decomposed using a set of plane waves propagating at different angles, and the electric field is projected onto a hemisphere 1 m away from the structure. The calculation was done using a built-in function called “farfield3d”, but could be more rigorously done following Ren *et al* [4]. To calculate the power flow from the complex electric field at each point on the hemisphere, the Poynting vector flux perpendicular to the hemispheric surface was calculated by

$${\boldsymbol{P}(r, \theta, \varphi,f)}_{\perp}=n\sqrt{\frac{\varepsilon_{0}}{\mu_{0}}}\left| \boldsymbol{E}(r, \theta, \varphi,f) \right|^{2}$$

where $n$ is the refractive index of a medium. The Poynting vector flux can be also called the power density having a unit of [W/m^2^]. We performed the calculation on the array with L = 1.4 $\mu$m, with two different sources using the same simulation setup as mentioned above. The data was extracted at $\lambda$ = 5.51 $\mu$m for the dipolar resonance, and $\lambda$ = 2.98 $\mu$m for the quadrupolar resonance. **Figure 2e** is the xz-cross section of the hemisphere at $\varphi$ = 0°. To compare power density between the dipolar and quadrupolar resonances, actual powers emitted by the local sources (ED and PEQ) were recorded in Watt as a function of frequency and used to normalize the power density at each position on the hemisphere.

- 1. **Q-factor calculation**

We utilized four time monitors around the corners of the gap (hot spot regions) to record electric and magnetic field data as a function of time. Then the signal was Fourier transformed into the frequency region. Spectra from individual time monitors were summed up and normalized to the source spectrum at each frequency. The full-width at half-maximum ($\Delta\omega$) was found from the dipolar and quadrupolar resonance peaks. Q-factor was estimated as Q = $\omega/\Delta\omega$.

- 1. **Cavity mode volume calculation**

Although the exact procedure for calculating mode volume in plasmonic cavities is a subject of current research, we adapted the equation defined in [5, 6] which was derived from the electric field energy density in the cavity. Since molecules could only reside in the air region of the nanorod arrays, while high field intensity also occupies the dielectric layer, we updated the equation for effective mode volume to integrate over these two regions separately following the preprint from our group [7]:

$$\mathcal{V}= \frac{\int_{\mathcal{V}1} \varepsilon_{1}\left| E \right|^{2}d\mathcal{V}+ \int_{\mathcal{V}2} \varepsilon_{2}\left| E \right|^{2}d\mathcal{V}}{{max}_{\mathcal{V}1}(\varepsilon_{1}\left| E \right|^{2})}$$

where $\mathcal{V}1$ refers to the volume surrounding the Au nanorods extending to 500 nm above the dielectric layer with refractive index of 1.45 for PMMA and 1 for air. The calculation wasn’t affected by the integration volume in air after 500 nm, because most of the $\left| E \right|^{2}$is concentrated near the structure well below 500 nm. The $\mathcal{V}2$ is the 40 nm of SiO_2_ dielectric layer with its frequency dependent dielectric function $\varepsilon_{2}$. The effective mode volume of quadrupolar plasmonic mode was calculated by exciting the nanorod array of **L** = 2.75 $\mu$m with PEQ, while that of the dipolar plasmonic mode was calculated by exciting the nanorod array of **L** = 1.3 $\mu$m with ED, which had their cavity frequencies tuned to the C=O vibration. We acknowledged that $\mathcal{V}$ is a rough estimate based on the un-interpolated electric field data recorded after applying apodization to cut the initial signal of the point sources.

1. **Experimental setup**
   1. **Fabrication**

We fabricated gold nanorods array using electron beam lithography on thin SiO_2_ on top of gold film deposited by plasma-enhanced chemical vapor deposition and electron beam deposition, respectively. The 200 nm of PMMA layer was coated by the spin-coating. Nanorod length (**L**) was modulated from 1.1 ~ 1.5 $\mu$m to tune DPL, and from 2.3 ~ 3.0 $\mu$m to tune QPL to PMMA molecular vibration. After the lithography, substrates were soaked in MIBK:IPA (1:3) solution for 60 seconds for the development. 100 nm of gold was evaporated to make the gold nanorods, then the substrates were soaked in Acetone for the lift-off. For VSC experiments, 200 nm of PMMA was spin-coated on top of substrates.

- 1. **FTIR measurement**

The infrared spectra were acquired on a Shimadzu AIM-8800 automatic infrared microscope using a 15x objective (numerical aperture of 0.3). A bare 40 nm thick SiO_2_ on top of 100 nm Au substrate was used as a reference prior to taking spectra of the plasmonic arrays. The control PMMA spectra were obtained on the 200 nm PMMA coated on 40 nm thick SiO_2_ on top of 100 nm Au film. The light source was unpolarized and the 32 spectra were averaged on the same sampling area. Measured absorbance from the microscope was converted to absorption using, $Absorption=1-{10}^{\left( -Absorbance \right)}$.

- 1. **Two-coupled oscillator model**

We adapted the two-coupled oscillator equation that was derived in many other literatures [8-10]. The experimental spectra were fitted to the equation for the extinction cross-section ($\sigma_{ext}$) of plasmon oscillation coupled with a molecular oscillation. The model assumed that the plasmon cross-section is much larger than that of the vibrational mode, so that the direct driving of the vibration by the external field can be ignored.

$$\sigma_{ext} \propto F_{pl}*\omega*Imag\left( \mu_{pl} \right)$$

$$\mu_{pl}= \frac{F_{pl}(\omega_{vm}^{2}-\omega^{2}-i\omega\gamma_{vm})}{\left( \omega_{vm}^{2}-\omega^{2}-i\omega\gamma_{vm} \right)\left( \omega_{pl}^{2}-\omega^{2}-i\omega\gamma_{pl} \right)- \omega_{vm}\omega_{pl}g_{N}^{2}}$$

Where $F_{pl}$ is the direct driving of plasmon by external field; $\mu_{pl}$ is the oscillating dipole of the plasmon; $\omega_{pl}$, and $\omega_{vm}$ is the plasmon and vibrational frequency; $\gamma_{pl}$, and $\gamma_{vm}$ is the plasmon and molecular linewidth; $g_{N}$ is the collective coupling strength. As pointed out in *Leng et al*. [11], $g_{N}$ is equal to the on-resonant vacuum Rabi frequency, which differs by a factor of 2 from the definition of $g_{N}$ in some cases in the literature [12]. Therefore, $g_{N}$ = 2$\sqrt{N}g_{s}$, where $g_{s}$ is the coupling strength of a single molecule with one cavity mode.

**Fig. S3** Calculated far-field dispersion of DPL-PMMA system as a function of angle of incidence ($\boldsymbol{\theta}^{\circ}$). Same calculation method used to obtain **Figure 4a** in the main text was used, but with the array of **L** = 1.3 $\mu$m which tunes dipolar plasmon to PMMA C=O stretch. The intensity in this plot shows electric field intensity and scaled arbitrary to emphasize relative polariton peak intensities over the background.


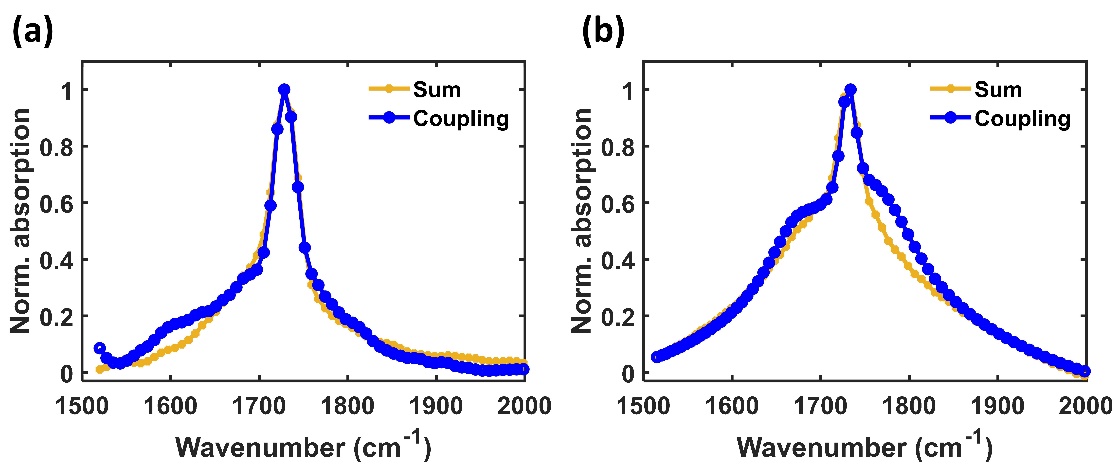


**Fig. S4** (a) Experimental and (b) calculated coupling and numeric sum spectra of QPL-PMMA resonant system. Each spectrum was normalized to the maximum, which was the intensity at molecular frequency, to compare broadening of the shoulder peaks.

- 1. **Estimation to the number of C=O bonds in a single unit cell**

The C=O density deposited on the substrate can be calculated by dividing the PMMA density (1.19 g/cm^3^) by the PMMA monomer weight (100.12 g/mole) and multiplying by Avogadro’s number, yielding 7.16 X 10^21^ (C=O)/cm^3^. In a single unit cell of periodicity $P$ = 1.6 $\mu$m and PMMA thickness of 200 nm, the volume occupied by the polymer was calculated by subtracting the volume of two nanorods with **L** = 1.3 $\mu$m from the 1.6 X 1.6 X 0.2 $\mu$m^3^. Multiplying the C=O density to the resulting volume yields the total number of C=O in a single unit cell, which was 3.58 X 10^9^. This number is on the highest end of our estimation ignoring the concentration of deposited solution and the disorder of the film. To estimate the number of coupled C=O (*N*) on DPL-PMMA substrate, we used theoretical $g_{s}$= 1 X 10^-5^ eV estimated from $V_{eff}$ obtained from DPL-PMMA simulation. The coupling strength between an electromagnetic cavity and a single molecular transition can be described as [[12](#_ENREF_12), [51](#_ENREF_51), [53](#_ENREF_53), [54](#_ENREF_54)]

${\hbar g}_{s}= \sqrt{\frac{\hbar\omega_{pl}}{2\varepsilon\mathcal{V}_{eff}}}\hat{\boldsymbol{e}}\cdot\vec{\boldsymbol{d}}$

where $\varepsilon$ is the permittivity of the medium at the location of the emitter, $\mathcal{V}_{eff}$ is the effective cavity mode volume, $\hat{\boldsymbol{e}}$ is the cavity field polarization direction, and $\vec{\boldsymbol{d}}$ is the vibrational transition dipole moment. A typical dipole moment of 1 debye associated with the C=O bond in PMMA [[47](#_ENREF_47)] was used for calculation. The equation for *N* used was,

$$N=\frac{g_{N}^{2}}{4g_{s}^{2}}$$

where $g_{N}$ = 73 cm^-1^ from two-coupled oscillator fit to DPL-PMMA resonant spectrum was used. Therefore, the estimated number of coupled molecules is 2.0 X 10^5^, which accounts for only 0.0056 % of the total C=O in a single unit cell. Additionally, we estimated the number of coupled molecules in QPL-PMMA system using $g_{N}$ = 103 cm^-1^ predicted by the computational near-field spectrum. The numerically calculated effective cavity mode volume for QPL mode was $\mathcal{V}_{eff}=$8.5 X 10^-21^ m^3^, which yields ${\hbar g}_{s,qpl}\boldsymbol{\approx}$ 6.8 $\mu$eV. Using the same equation for number of coupled molecules, it was estimated that that about 8.1 X 10^5^ C=O bond is participating in the VSC of QPL-PMMA resonant system in the near-field.

1. **References**

[1] E.D. Palik, *Handbook of Optical Constants of Solids*. Vol. 1-3. 1998: Academic Press.

[2] X. Zhang, J. Qiu, J. Zhao, X. Li and L. Liu, "Complex refractive indices measurements of polymers in infrared bands," *Journal of Quantitative Spectroscopy and Radiative Transfer*, vol. 252, no. 107063, 2020, <https://doi.org/https://doi.org/10.1016/j.jqsrt.2020.107063>.

[3] B. Liang, M. Bai, H. Ma, N. Ou and J. Miao, "Wideband Analysis of Periodic Structures at Oblique Incidence by Material Independent FDTD Algorithm," *IEEE Transactions on Antennas and Propagation*, vol. 62, no. 1, pp. 354-360, 2014, <https://doi.org/10.1109/TAP.2013.2287896>.

[4] J. Ren, S. Franke, A. Knorr, M. Richter and S. Hughes, "Near-field to far-field transformations of optical quasinormal modes and efficient calculation of quantized quasinormal modes for open cavities and plasmonic resonators," *Physical Review B*, vol. 101, no. 20, pp. 205402, 2020.

[5] S. Huang, T. Ming, Y. Lin, et al., "Ultrasmall Mode Volumes in Plasmonic Cavities of Nanoparticle-On-Mirror Structures," *Small*, vol. 12, no. 37, pp. 5190-5199, 2016, <https://doi.org/https://doi.org/10.1002/smll.201601318>.

[6] O. Painter, R.K. Lee, A. Scherer, et al., "Two-Dimensional Photonic Band-Gap Defect Mode Laser," *Science*, vol. 284, no. 5421, pp. 1819-1821, 1999, <https://doi.org/doi:10.1126/science.284.5421.1819>.

[7] Z.T. Brawley, J. Yim, S. Pannir-Sivajothi, Y. Poh, J. Yuen-Zhou and M. Sheldon, "Sub-wavelength chemical imaging of a modified reaction due to vibrational strong coupling," *ChemRxiv*, vol. no. 2023, <https://doi.org/10.26434/chemrxiv-2023-gdmxl>.

[8] Z.T. Brawley, S.D. Storm, D.A. Contreras Mora, M. Pelton and M. Sheldon, "Angle-independent plasmonic substrates for multi-mode vibrational strong coupling with molecular thin films," *The Journal of Chemical Physics*, vol. 154, no. 10, pp. 104305, 2021, <https://doi.org/10.1063/5.0039195>.

[9] M. Pelton, S.D. Storm and H. Leng, "Strong coupling of emitters to single plasmonic nanoparticles: exciton-induced transparency and Rabi splitting," *Nanoscale*, vol. 11, no. 31, pp. 14540-14552, 2019, <https://doi.org/10.1039/C9NR05044B>.

[10] X. Wu, S.K. Gray and M. Pelton, "Quantum-dot-induced transparency in a nanoscale plasmonic resonator," *Opt. Express*, vol. 18, no. 23, pp. 23633-23645, 2010, <https://doi.org/10.1364/OE.18.023633>.

[11] H. Leng, B. Szychowski, M.-C. Daniel and M. Pelton, "Strong coupling and induced transparency at room temperature with single quantum dots and gap plasmons," *Nat. Commun.*, vol. 9, no. 1, pp. 4012, 2018, <https://doi.org/10.1038/s41467-018-06450-4>.

[12] G. Khitrova, H.M. Gibbs, M. Kira, S.W. Koch and A. Scherer, "Vacuum Rabi splitting in semiconductors," *Nature Physics*, vol. 2, no. 2, pp. 81-90, 2006, <https://doi.org/10.1038/nphys227>.
